# Understanding Student Characteristics in the Development of Active Learning Strategies

**DOI:** 10.1007/s40670-022-01550-9

**Published:** 2022-04-30

**Authors:** Seema Mehta, Casey P. Schukow, Amar Takrani, Raquel P. Ritchie, Carol A. Wilkins, Martha A. Faner

**Affiliations:** 1 College of Osteopathic Medicine, Detroit Medical Center, Michigan State University, Detroit, MI 48201 USA; 2 College of Osteopathic Medicine, Macomb University Center, Michigan State University, MI 48038 Clinton Twp, USA; 3 College of Osteopathic Medicine, Michigan State University, East Lansing, MI 48824 USA

**Keywords:** Active learning, Medical school, Biochemistry, Motivated strategies for learning questionnaire

## Abstract

Student evaluations after non-lecture, active learning sessions at Michigan State University, College of Osteopathic Medicine, have yielded bipartite responses with one group responding favorably and the other group negatively. The purpose of this study was to understand the characteristics, motivation, and learning strategies of medical students that find value in, appreciate, and perceive active learning sessions to be beneficial and those of students that do not. We generated a survey, based on the Motivated Strategies for Learning Questionnaire (MSLQ), that included questions regarding overall student motivations for learning, learning strategies that students employed throughout medical school, and their perceptions of active learning. Following an active learning session on hyperammonemia, we administered the modified MSLQ survey. Using the results of this survey, we validated the modified MSLQ and identified correlations between student characteristics and their perception of the active learning session. We found that, in general, students with high task value, intrinsic goal orientation, self-efficacy for learning and performance, and control of learning beliefs felt more positively about their experience in the active learning session. Understanding the characteristics, motivations, and learning strategies that help students find value in active learning sessions will help medical educators develop future curricular material so that these exercises will better engage and be more effective with a greater number of learners.

**Supplementary Information:**

The online version contains supplementary material available at 10.1007/s40670-022-01550-9.

## Introduction

Over the past decade, undergraduate medical education has witnessed a major shift in focus from teacher-centered education models towards active, learner-centered instruction [1, 2]. Often presented as a radical change from traditional instruction, active learning is generally defined as any instruction that engages students in the learning process rather than passively taking it in [2]. This form of teaching allows students to engage in meaningful learning activities that encourage self-reflection about the content of one’s own education [3]. Active learning has been found to be effective in maximizing learning, improving collaboration with peers, and optimizing the practice of evidence-based medicine [1, 4–6]. It allows students to remain at the forefront of the learning process and encourages higher-order thinking through practices such as problem solving, case-based learning, and self-monitored learning [7]. Active learning sessions are effective at fostering the self-reflection that medical students engage in while learning and prepares them for the management of complex clinical cases through the translation of biomedical knowledge into clinical practice [8, 9]. In addition, medical professionals are expected to engage in life-long learning which requires two cardinal tenets of the active learning process: self-regulation and monitoring of learning [8]. The Commission on Osteopathic College Accreditation (COCA) board and the Liaison Committee on Medical Education (LCME) have standards that include a requirement for self-directed learning and independent study, which are both examples of active learning, in their medical curriculums [10, 11]. As such, reform efforts in medical education have also acknowledged the importance of this necessary shift in learning.

Recognizing the value in active learning, the biochemistry faculty at Michigan State University College of Osteopathic Medicine (MSU COM) began to design sessions that use this mode of learning into the primarily lecture-based pre-clerkship curriculum. After debuting these sessions, qualitative student feedback was sought. Students’ responses were dichotomous. Some enjoyed and saw value in the sessions while others disliked and viewed the sessions as a waste of time. We hypothesized that these groups of students with opposing viewpoints may have certain characteristics about them as learners, related to how they felt about these sessions. If so, a greater understanding of the characteristics, motivations, and learning strategies of students who find value in active learning sessions, as well as those who do not, would be particularly useful for attempts at optimizing these sessions for all medical students.

The Motivated Strategies for Learning Questionnaire (MSLQ) is a tool that was developed by Pintrich et al. at the University of Michigan to measure academic motivation and learning strategies among students [12]. As a self-report instrument, the MSLQ has proven to be a reliable and validated study tool that may be adapted to various student populations and contexts [13, 14]. It consists of two scales, including motivation and learning strategies, that are scored on a 7-point Likert measure [12]. In a study by Soemantri et al., a systematic search of 401 journal articles revealed that the MSLQ was the most effective questionnaire for measuring the reflective learning of medical students [8].

This study uses a modified form of the MSLQ as an instrument to better understand the motivational beliefs and learning strategies of medical students at MSU COM and explore correlations between those attributes and how they perceived an active learning session. This understanding will allow medical educators to optimize activity sessions to engage students that do not perceive active learning sessions to be beneficial, while further encouraging the students that do. In doing so, this study fills an important gap in the literature on this topic and provides biomedical educators with the additional guidance to create an optimal learning environment in medical school for all learners.

## Materials and Methods

### Context

**Fig. 1:**
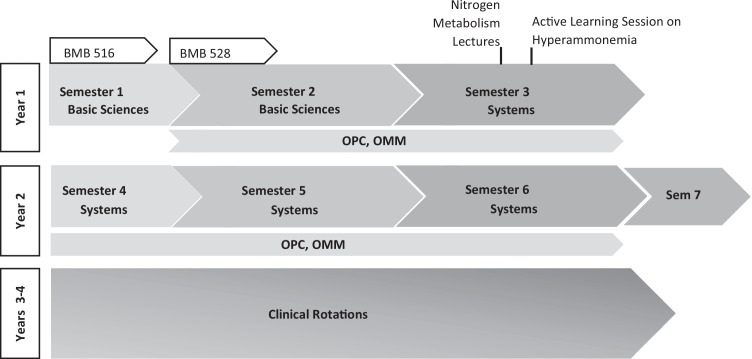
MSU COM curriculum. The 4-year curriculum is broken into 2 years of pre-clerkship and 2 years of clinical rotations. The summer semesters (1 and 4) are 10 weeks long. Semesters 2, 3, 5, and 6 are approximately 15 weeks long and semester 7 is a short transitional semester between pre-clerkship and clerkship. Osteopathic Patient Care (OPC) and Osteopathic Manipulative Medicine (OMM), are two clinical courses that run longitudinally throughout the first 2 years. Basic science courses, including two biochemistry and molecular biology courses, occur in the first two semesters. Biochemistry content is also integrated into systems courses; for example, nitrogen metabolism is covered during lectures in the genitourinary course. Approximately 2 weeks after lectures on nitrogen metabolism, the students participate in an active learning session on hyperammonemia

This study was conducted at MSU COM, which offers a 4-year medical program across three campuses to a class of nearly 300 students. The East Lansing campus accommodates approximately 200 students, while the Detroit Medical Center and Macomb University Center host about 50 students each. The pre-clerkship curriculum (Fig. 1) is primarily lecture-based with some small-group discussion sessions, patient presentations, and unguided individual study. At the end of semester 6, MSU COM students take the Comprehensive Osteopathic Medical Licensure Examination Level One (COMLEX-USA Level 1) evaluation and subsequently transition into the clerkship curriculum/clinical rotations. The biochemistry, molecular biology, and genetics curriculum within the pre-clerkship program consists of two main courses totaling three credits. In addition, there are several lectures and activities integrated into other courses spanning the 2 years. Overall, the content is delivered primarily via lecture with some active learning sessions (Table 1). Appendix 1 provides more specific information about the types of active learning activities listed in Table 1.

**Table 1:** Delivery methods of biochemistry, molecular biology, and medical genetics content in MSU COM pre-clerkship courses

**Course**	Active learning*	Lecture*	Patient presentation*
Metabolic Biochemistry	5	10	
Molecular Biology & Medical Genetics	2	28	1
Pathophysiology		8	
Endocrine		6	
Genitourinary	1	4	
Cardiology		2	1
Ethics	1		
Osteopathic Patient Care	2		1

^*^Active learning consists of any case-based sessions, flipped classrooms, or student-driven inquiry session that is not a lecture. Lectures are traditional in format but may include active components like student polling and group problem-solving. Patient presentations involve a visit to the classroom from a patient who talks about their medical condition and life-experience with the condition

Over the course of several years, MSU COM biochemistry faculty added active learning sessions to the curriculum to improve students’ attitudes toward their learning, retention of curricular material, critical thinking skills, and self-direction and collaboration. Most of these newly developed sessions took place within the two Biochemistry, Molecular Biology, and Genetics courses (Fig. 1, Table 1, and Appendix 1). However, student feedback from these sessions early in the curriculum yielded opposing viewpoints, with some liking them while others disliking them. To improve these sessions, we sought to gain some understanding of the characteristics of the students using the MSLQ vehicle. The main reason for choosing to administer the MSLQ survey following the session on hyperammonemia was the latter’s placement late in the series of active learning sessions involving the biochemistry faculty. The choice was based, therefore, not so much on the subject matter but simply because it was one of the last opportunities in which the biochemistry faculty had a direct encounter with the students in an active learning session (Fig. 1, Table 1, and Appendix 1).

The hyperammonemia session took place approximately 2 weeks after four lectures on nitrogen metabolism during the students’ semester three genitourinary course (Fig. 1). The coverage included digestion and absorption of proteins and amino acids, nitrogen balance, amino acid metabolism particularly in terms of the urea cycle and fate of the carbon skeletons, and inter-organ relationships of nitrogen fixation or removal. This provided students time to assimilate the information before applying it to a patient case. As with most of the other active learning sessions described in Appendix 1, the class was divided into six classrooms (~50 students/classroom with one faculty) and the students worked collaboratively in teams of eight within their rooms. The session begins with a case presentation on a patient with hyperammonemia. Students then collaborate within their teams to identify key words most appropriate for a literature search on a recent review article on hyperammonemia diagnosis and management (~5 min); the classroom then assembles as a whole, and teams present their work product. Using the common top five key words, students then break out into teams again to identify the best review articles (~20 min); the classroom reassembles as a whole, and teams present their findings. Within certain set boundaries (e.g., article published within the last 5 years), the article by Haberle et al., outlining guidelines for the diagnosis and management of urea cycle disorders has come up as one of the top five in most of the selections identified by the student teams [15]. On this basis, the faculty declares it as the “gold standard” and students break out into teams to read the article, guided by a set of questions on the etiology, symptoms, biochemical basis, diagnosis, and management of the condition. For this reading (~30 min), students were encouraged to adopt a strategy of “divide-and-conquer,” with one member of the team responsible for etiology, another responsible for diagnosis and testing, etc. Team members share their individual findings and each team then constructs a summary of their collective work product, which is submitted for grading. Finally, the classroom reassembles, and teams can present and share their summary. Despite some of the negative feedback (in addition to positive) that we received on this exercise, we have continued to offer it (while continually seeking to improve it) because it provides a valuable opportunity for the students to work as a team, synthesize and summarize scientific literature, and present their findings. These are all valuable skills for future physicians and are difficult to teach in a traditional lecture environment.

### Data Collection

The participants in this study were first-year osteopathic medical students (OMS-I) from the 2020, 2021, and 2022 graduating classes. Following completion of the hyperammonemia session, students were invited to return a questionnaire (either online or via scantron), which took 15–20 min to complete and was offered over a 2-week period. The questionnaire informed students of the study, in which participation was voluntary and anonymity was guaranteed. Of the ~900 students (300 in each class) that were invited to participate, a total of 213 questionnaires were collected and 192 students answered all the questions: 93 questionnaires in 2017, 38 in 2018, and 61 in 2019. No identifying or demographic information was collected from the participants. No potential risk or harm to students participating in this study was expected and approval was obtained from the Michigan State University Institutional Review Board on Human Subjects (IRB # × 14-185e, February 2014).

### Instrument

**Fig. 2:**
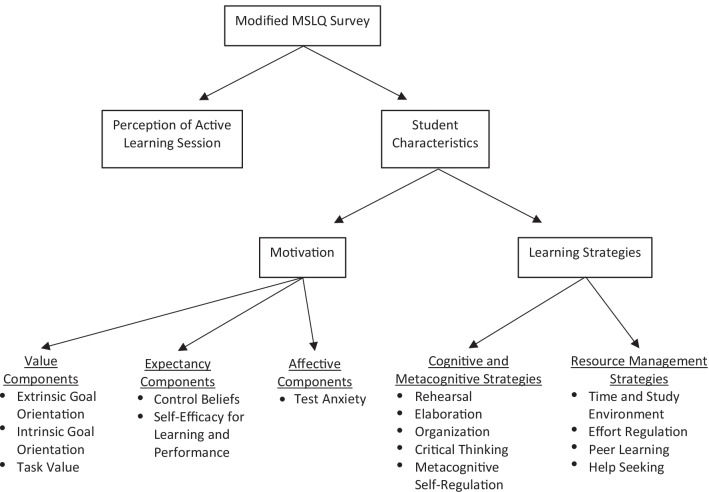
The components and associated subscales of the modified MSLQ survey administered to study participants. The survey contained 89 items divided into two sections: student perception of the hyperammonemia active learning session (student perception of active learning sessions such as hyperammonemia?); student characteristics in terms of motivation and learning strategies. Subscales listed under motivation and learning strategies are described in Pintrich et al. [12]

The original MSLQ consists of 81 survey items divided into nine learning strategy subscales and six motivation subscales [12]. Since the MSLQ subscales were designed to fit the needs of any study, they were appropriately adopted for our current analysis with modifications to reference the MSU COM curriculum. Eight additional items were added to measure student perception of the hyperammonemia session and active learning in general (labeled as “Perceptions of Active Learning” in Fig. 2). Seven of these questions specifically address student perceptions of the hyperammonemia session and one asks about inclination to attend class in-person for active versus lecture style format. The purpose of these questions was to measure student perception so that we could investigate if there is a correlation between those perceptions and the student characteristics measured by the MSLQ. This study also elected to use the 7-point Likert scoring scale of the original MSLQ, with 1 corresponding to “not at all true of me” and 7 indicating “very true of me.” The modified questionnaire is provided as Appendix 2 of this article and Table 2 displays the eight additional questionnaire items used in the present study along with a sample of two of the modified MSLQ items. The final survey was comprised of 89 items that were divided into two sections: the first assessed student perceptions of the hyperammonemia session and the second assessed student characteristics. The student characteristics section was further divided to assess motivation and learning strategies each of which contained subscales as depicted in Fig. 2. The value components subscale measures extrinsic goal orientation (motivation that stems from external sources), intrinsic goal orientation (motivation that stems from internal reasons), and task value (how interesting, useful, or important a task seems). The expectancy components subscale is made up of items assessing control beliefs (belief that outcomes are contingent on one’s own effort) and self-efficacy for learning and performance (self-appraisal of one’s ability to accomplish a task). The affective components subscale is made of a single component on test anxiety. The cognitive and metacognitive strategies subscale measures learning strategies and is composed of rehearsal (reciting or naming items from a list), elaboration (paraphrasing, summarizing, creating analogies), organization (clustering, outlining, and selecting the main idea), critical thinking (applying previous knowledge to a new situation), and metacognitive self-regulation (awareness, knowledge, and control of cognition). The other learning strategies subscale is resource management strategies and measures time and study environment (scheduling, planning, time management), effort regulation (ability to focus when distracted or uninterested), peer learning (collaboration with peers), and help seeking (identify gaps in knowledge and those who can assist). For a complete description of the subscales, please see Pintrich et al. [12].

**Table 2:** Sample questions from the modified MSLQ*

1. This session has improved my ability to search for relevant journal articles
2. This session reinforced important concepts on the topic of nitrogen metabolism (ammonia and urea) previously introduced in [the genitourinary system course]
3. This session improved my understanding of how basic science content (urea cycle) forms the basis for clinical application (hyperammonemia)
4. Reading the gold standard article as a team was an efficient way to learn the necessary information
5. Writing a summary as a team helped me in understanding content as well as in developing clarity of thought
6. I felt a greater sense of social support from my classmates during this session than in a traditional lecture
7. In general, I feel an active learning session (like the one I experienced today) is a more effective way than lectures to learn the required material
8. In general, I would be more inclined to attend class in person if the time was used for group activities or problem solving rather than a lecture presentation
19. The most important thing for me right now is improving my *overall chances of a desirable residency match*, so my main concern in this curriculum is getting good grades Original MSLQ: The most important thing for me right now is improving my *overall grade point average*, so my main concern in this class is getting a good grade
38. I want to do well in this curriculum because it is important to show my ability to *prospective residency directors* Original MSLQ: I want to do well in this class because it is important to show my ability to *my family, friends, employer, or others*

^*^Shown are eight added questionnaire items that measure student perceptions of active learning and two original MSLQ items with that have been slightly modified to reflect a medical school context

### Exploratory Factor Analysis

Using the statistical analysis software SPSS 25.0.0.0 (SPSS Inc., Chicago, IL), we first applied an exploratory factor analysis (EFA) on the items intended to measure student characteristics (9–89) to gain a general understanding of the patterns in the data [16]. EFA identifies common factors that help explain a measured variable [16]. The original dataset was divided into two, and an EFA was performed on half of the dataset (*n* = 96), which included half of the data from each cohort year (the classes of 2020, 2021, and 2022). A principal axis factor estimator was used to extract the factors from this dataset and help determine coefficients to relate factors with multiple variables [16]. An oblique (quartimin) rotation was chosen for the EFA as the theorized model predicted a correlation between the instrument’s items. The nine factors predicted by the EFA and their pattern coefficients are in Appendix 3. Only items with a pattern coefficient of > 0.4 were considered.

**Fig. 3:**
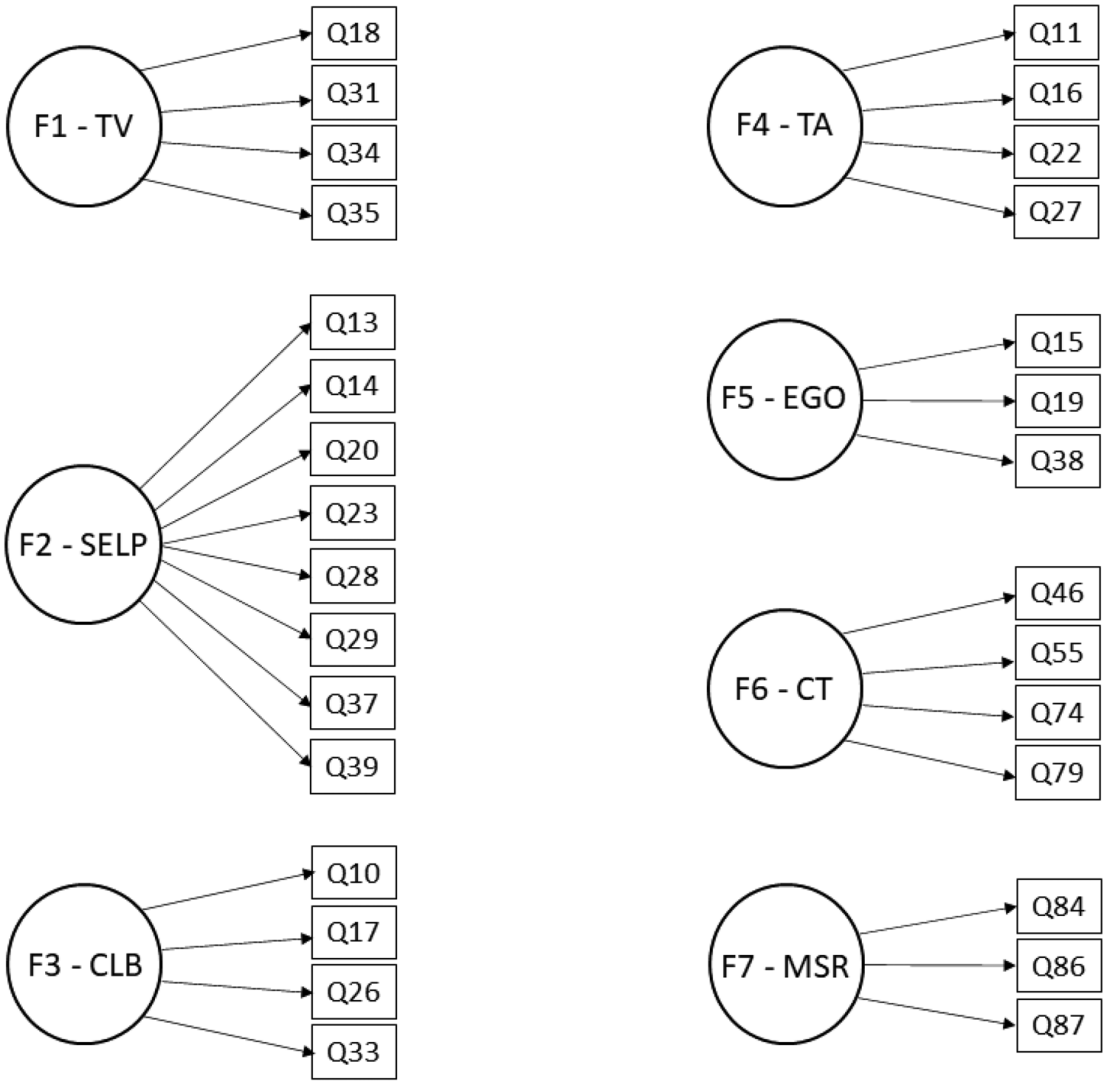
MSLQ-MSU COM. The seven factors used in our model based on factor loading as determined by the EFA as well as theoretical considerations. The seven factors are labeled F (for factor) followed by their respective factor number. An abbreviation that indicates what the factor measures is given next to the factor number and is defined as follows: F1, Task value; F2, Self-efficacy for learning & performance; F3, Control of learning beliefs; F4, Test anxiety; F5, Extrinsic goal orientation; F6, Critical thinking; F7, Metacognitive self-regulation. The question numbers that loaded on a given factor are indicated in boxes and connected to their assigned factor with arrows

The EFA analysis, and theoretical considerations (the original MSLQ sub-scales), informed the construction of the final seven factor model (Fig. 3) that we have named “MSLQ MSU COM” [12]. All the factors were made up of three or more questions. All the questions making up the factors were designed to test that factor in the original MSLQ. For example, the task value (TV) factor was made up of four questions (numbers 18, 31, 34, 35) all designed to measure task value. There were other questions that indicated covariance with items in the final factors that were omitted on the basis that they did not logically group with the other questions in that factor. For example, questions 46, 55, 74, and 79 had strong covariance and were all meant to measure critical thinking. The EFA also identified questions 60, 64, 68, and 72 as covarying with those questions but they did not measure similar characteristics (Appendix 3).

### Confirmatory Factor Analysis

Based on the results from the EFA, a confirmatory factor analysis (CFA) was then applied to the other half of the dataset (*n* = 96) using the seven-factor model defined from the EFA (Fig. 3). This analysis is performed to validate the seven-factor model proposed by the EFA by confirming the relationship between the items measured on the MSLQ and the factors produced by the EFA [16]. Computations were completed using the AMOS module of SPSS 25.0.0.0 (SPSS Inc., Chicago, IL). The CFA was specified using the seven-factor model, with 30 items. The CFA demonstrated a reasonable model fit as indicated by CMIN/d.f. = 2.047 (< 3.0 is considered a reasonable fit) and *p* < 0.05, while the other indices approached significant validity (*χ*^2^ = 800.5, d.f. = 391, CFI = 0.863, SRMR = 0.074). The comparative fit index (CFI) compares the fit of the model being tested with a model in which none of the variables is related. A CFI > 0.95 indicates that the tested model fits the data well so our model fell just short of that [16]. The standardized root-mean-square residual (SRMR) should be < 0.08 to be acceptable and our model did come in under 0.08 [16]. Using the coefficient (Cronbach’s) alpha, which measures internal consistency and thus how closely items within a dataset are related, all seven factors exceeded the 0.70 cutoff for reasonable acceptance of the factor. Statistically significant co-variances were demonstrated between Test Anxiety and two additional factors: Self-Efficacy for Learning & Performance (TA -0.248, *p* = 0.02) and Control of Learning Beliefs (− 0.175, *p* = 0.049) which was predicted in the EFA. CFA indices and standard regression weights for each of the seven factors constructed by the EFA are in Appendix 4.

Similar analyses have been conducted before. Pintrich et al., the original developers of the MSLQ, applied a confirmatory factor analysis to the original questionnaire upon its publication [17]. They determined that the questionnaire demonstrated good factor structure and that the MSLQ shows reasonable predictive validity to the actual course performance of students. A study evaluating the questionnaire among Iranian high school students found that it was reasonably reliable, with an EFA determining strong construct validity [18]. A Turkish study, which looked at both elementary and high school students, applied a CFA to the questionnaire items, and deduced that the models relevant to the motivation scale and learning strategy subscale demonstrated an acceptable fit [19]. Another study evaluating student perceptions and motivations in asynchronous online learning environments (AOLE) considered all MSLQ items and determined that, within AOLE settings, the questionnaire demonstrated poor factor structure [20]. The original authors of the MSLQ instrument intended to measure (generally) the types of learning strategies and academic motivation used by undergraduate students. Our derived factors support this goal, but specifically within the context of active learning environments. This speaks to the importance of adapting the original MSLQ to reflect specific learning mediums, and subsequently conducting a factor analysis on all items to determine instrument validity.

## Results

### Approach

Our overall approach to data analysis included screening of the datasets from all three class years for missing values and errors in data entry. Statistical analysis began with an exploratory factor analysis (EFA) to define valid factors in our survey. Once we established a model from this analysis, we performed a confirmatory factor analysis (CFA) to ensure validity of the model and of the questionnaire’s use. These analyses provide confidence to the researcher employing the instrument and serve as markers of validity for future studies that may utilize the same modified instrument [16]. We were able to validate the use of our model, MSLQ MSU COM, to measure task value (TV), self-efficacy for learning and performance (SELP), control of learning beliefs (CLB), test anxiety (TA), extrinsic goal orientation (EGO), critical thinking (CT), and meta-cognitive self-regulation (MSR) in our students. TV refers to student evaluation of how important or useful a task is (“What do I think of this task?”), while SELP measures one’s ability and confidence to master/perform a task (“I believe I will do well in this class”) [12]. CLB represents students believing that positive outcomes are due to their own effort and control within a learning environment (“If I study in appropriate ways, then I will be able to learn this material”) [12]. TA has two components: a worry component with negative thoughts (“When I take a test, I think about how poorly I will do”), and an emotional component related to physiologic aspects of anxiety (“I feel my heart beating fast when taking an exam”) [12]. EGO involves students’ concerns with malleable ends to their learning, such as grades, rewards, and financial gain (“I am most concerned with getting a good grade in this class”) [12]. CT refers to how students apply previous knowledge to solve new problems or reach decisions (“I try to apply ideas of my own when learning about new material”) [12]. Finally, MSR applies student awareness, knowledge, and control of cognition through thought planning, monitoring, and regulating (“When learning new material, I make up questions to help focus my thoughts”) [12]. Following definition and confirmation of the factors that our instrument could test, series of bivariate Pearson coefficient and ANOVA analyses were completed to identify correlations between questions specific to student motivations, learning strategies, and perceptions of the hyperammonemia active learning session and active learning in general.

### Characteristics of MSU COM Students on Average

**Fig. 4:**
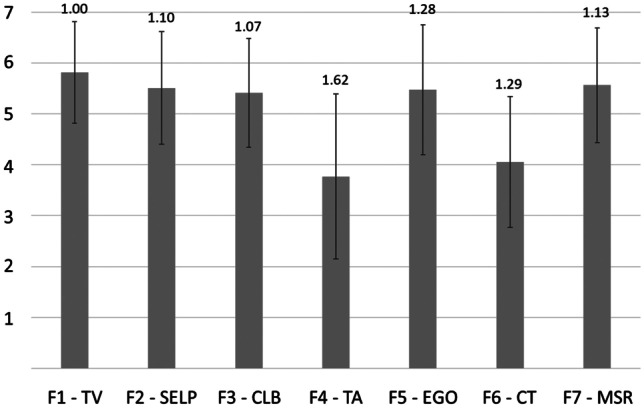
Characteristics of MSU COM Students for the seven factors measured in the MSLQ MSU COM survey. The data are expressed as means (height of the bar) and standard deviations (error bars and values above them) of students’ responses for the seven factors: TV, Task value; SELP, Self-efficacy for learning & performance; CLB, Control of learning beliefs; TA, Test anxiety; EGO, Extrinsic goal orientation; CT, Critical thinking; MSR, Meta-cognitive self-regulation

MSLQ MSU COM was scored for each respondent by obtaining the mean of the items that make up each factor as has been described in the use of the original MSLQ [12]. To get a feel for the characteristics of MSU COM students, the respondents’ scale means and standard deviations were calculated (Fig. 4). Students scored on the higher end of the scale (closer to very true of me) on the scales measuring task value, self-efficacy for learning and performance, control of learning beliefs, extrinsic goal orientation, and meta-cognitive self-regulation. Students scored on the middle of the scale for test anxiety and critical thinking. This indicates that our students may need help with critical thinking. Active learning sessions like the hyperammonemia session should help them practice using prior knowledge to solve problems.

### Pearson Correlations

To evaluate whether the way that students answered questions on the student characteristics portion of the survey had any correlation to how they answered the questions on the perception of active learning portion, we calculated Pearson correlation coefficients. The coefficient analysis was performed for all eight of the active learning questions with the seven factors from the MSLQ MSU COM model. In addition, we performed the analysis for the eight active learning questions with the student characteristic questions that did not co-vary with other items to comprise a factor. Even though those items did not group into a factor, they can still provide some insight as individual questionnaire items.

**Fig. 5:**
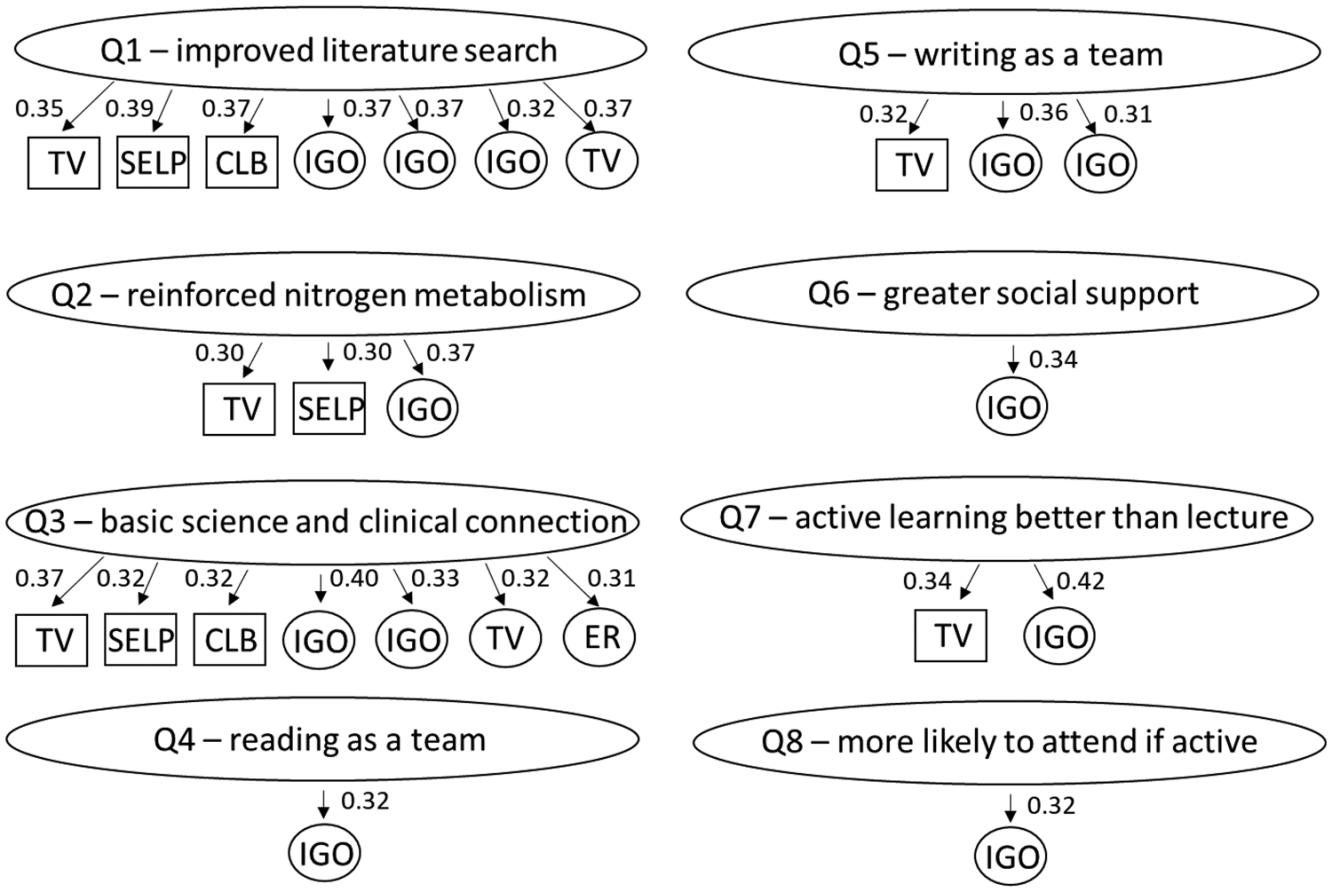
Correlation between perceptions of active learning session and student characteristics. Pearson correlations and *p*-values were calculated to assess associations between the eight active learning items (Table 2) with seven factors from the MSLQ MSU COM model (in boxes); and student characteristic questions that did not co-vary with other items to comprise a factor (in ovals). TV, Task value; SELP, Self-efficacy for learning & performance; CLB, Control of learning beliefs; IGO, Intrinsic goal orientation; ER, Effort regulation. Pearson correlation coefficients are noted next to the arrows. Only correlations with medium strength were noted. All correlations shown had *p*-values < 0.05

Pearson correlations and *p*-values were calculated to assess associations between the seven factors and eight active learning items (Fig. 5 and Appendix 5). The list of the eight questions on active learning can be found in Table 2. A moderate strength of association is presumed to have a Pearson coefficient value between 0.3 and 0.5, while a small strength of association is presumed to have a value between 0.1 and 0.3. We focused on results with moderately positive strengths of association and found those to be present between task value (factor 1) and questions 1, 2, 3, 5, and 7, self-efficacy for learning and performance (factor 2) and questions 1, 2, and 3, and control of learning beliefs (factor 3) and questions 1 and 3. This means that students who rated high on those characteristic scales also rated the active learning questions highly. We also found that some individual questions from the student characteristics portion of the survey had medium strength correlations with some of the questions on active learning. Question 9 measured intrinsic goal orientation and had a medium strength correlation with all eight of the active learning questions. Question 12 measured task value and had a medium correlation with questions 1 and 3. Question 30 measured intrinsic goal orientation and had a medium correlation with questions 1, 3, and 5. Question 32 measured intrinsic goal orientation and had a medium correlation with question 1. Question 56 measured effort regulation and had a medium correlation with question 3. All *p*-values for associations demonstrating medium strength were far below a cutoff of < 0.05, therefore demonstrating significance. No significant negative correlations were found.

## Discussion

One key result of our survey indicates that students who find value in the curriculum (task value, F1, Q12) recognize that several important objectives of the hyperammonemia active learning session were met, as indicated by their more favorable Likert scale choices (Fig. 5). Students who believe the overall MSU COM curriculum is interesting, important, and useful also attribute these values to the hyperammonemia session. These students, measuring high on the task value scale, further agree that active learning is more effective in achieving content understanding than through lectures. While task value may be innate to certain students, other students might struggle to find value in active learning sessions for several reasons. In a curriculum prioritizing lecture-based delivery, the underlying message some students may perceive is that lectures represent the optimal mode of content delivery. Thus, some students may identify active learning sessions as outliers and struggle to recognize the value of these sessions for their learning. Other students may simply be used to lectures and have difficulty realizing the value of other modes of delivery. We propose that this task value characteristic can be acquired or enhanced through more effective communication with students, both at the session level and at the curriculum level. Detailed communication at the session level of the objectives and mode of delivery (active learning versus lecture) will enable students to appreciate the importance and utility of the session towards their ability to apply concepts. Thoughtful design of the session such that the students will be solving an authentic problem has been shown to promote student interest [21]. Clear communication at the curriculum level conveys the importance of the active learning process in acquiring or enhancing the necessary skills of a physician.

Students with high self-efficacy for learning and performance recognized that the hyperammonemia session improved their ability to search the literature, reinforced nitrogen metabolism, and helped connect basic and clinical sciences (Fig. 5). These students, rating high on the self-efficacy scale, are confident in their skills to perform a given task. They are assured in their ability to learn content using a different methodology. This suggests that if we can help improve students’ confidence in their skills to perform session tasks, we may also help to improve their perceptions of active learning sessions. To improve student confidence in task performance, it is important to have clear objectives and instructions for the session and to communicate them in advance. Students will then know what tasks will be expected of them during the session. To make this connection, faculty knowledge of the sequence of skills acquired by students as they progress through the curriculum is also important to prevent or minimize incidences in which students are expected to perform unfamiliar tasks. For example, if most students are not experienced in executing applicable literature searches, session design would need to provide appropriate background prior to or during the session to ensure their ability to complete the session tasks. In fact, it has been shown that to enhance self-efficacy the learning environment should allow for collaboration and autonomy and should not make too many demands on learners which might result in negative emotions [22].

This study also found students who believe their efforts will result in positive outcomes (control of learning beliefs) indicated that the session improved their literature searching skills and helped them make connections between basic and clinical sciences (Fig. 5). These are students who take responsibility for their own learning, rather then rely on external factors like their teacher. This is the type of student one would expect to embrace active learning, which is inherently more student driven, rather than receiving content via a lecturer. Students who measure low in control of learning beliefs may be holding on to undergraduate study attitudes, in which students tend to be more reliant on their instructors in learning content. In a primarily lecture-based medical school curriculum, students may find it easier to continue to rely on instructors for their knowledge access. Increasing the number of active learning opportunities in all the pre-clerkship courses, along with clear communication upon their entry to medical school as to how active learning helps students acquire and enhance their skills as a physician, could help make the transition to control of their own learning.

As mentioned previously, our statistical model did not support factor grouping for many questions. When we performed correlation analysis using the questions that did not group into factors with the active learning survey questions, we identified some medium-strength correlations. One question (Q56), which measured effort regulation, had a medium-strength correlation with the question indicating the active learning session helped connect basic and clinical sciences. Certain students, even when they are uninterested or distracted, are still able to put out their best effort because they see the purpose of the active learning session in making the connection between those two domains. Three of those questions (Q9, Q30, Q32) that did not group into factors were intended to measure intrinsic goal orientation. In fact, Q9 had a medium correlation with all the active learning questions and, overall, had some of the strongest correlation coefficients that we observed in this study. Students who measure high in intrinsic goal orientation participate in the curriculum for reasons like challenge, curiosity, and mastery rather than to pass the course and their board exams, or become a physician to make money. Attending a session that is different from what they are used to and perceived as more difficult would not be as daunting to these students and, therefore, may allow them to enjoy and see value in such a session. Intrinsic goal orientation can be fostered in students by providing three psychological needs on which this type of motivation is dependent [23]. One strategy that appeals to these needs is to balance giving students more responsibility for their learning (autonomy) and providing structure and guidance (competence and relatedness). A well-designed session with clear expectations and opportunity for active participation can help achieve that balance. Additionally, focusing on the ideals and skills underlying a good physician is important to foster intrinsic goal orientation in our students. This appeals to their need for autonomy or that one is carrying out a task of their own choice since most medical students would say that they want to be good doctors. However, this focus often gets overshadowed by the stress of performing well on multiple choice examinations. Throughout their academic careers, many students have been learning based on their goal of excellent test performance. This goal does not change upon entry into medical school. However, changes in medical school programs are coming, with a shift to competency-based education and with USMLE Step 1 and NBOME COMLEX Level 1 moving to a pass/fail system. It will be of interest to see how these changes affect medical school curricula, and in turn, the way students learn. In part, this shift is occurring to align performance evaluation more closely with residency and beyond, where in addition to standardized tests (USMLE/COMLEX Step 3, in-service exams, licensure renewal exams), physicians are also evaluated through consistent faculty evaluations on clinical competency, medical knowledge, and physician–patient communication. Since the competencies and performance of physicians continue to be measured throughout their career, it is vital that their foundational characteristics, competencies, and skills be developed early on during medical school training.

Perhaps the greatest limitation in this study is the overall number of questionnaires we collected and deemed to be sufficient for the purposes of data gathering (*n* = 192/213). Approximately 300 new students attend MSU COM each year. Thus, we did not collect data on about 700 students, which could have provided a greater overall strength to the study [24]. One possible contributing factor is the time constraints that medical students face. The students were given a 2-week time frame to fill out this 20-min optional survey (either on paper or digitally), which may not seem like a major task from a medical educator’s standpoint. However, given the difficulty of the medical school curriculum and the time constraints imposed on them from classes, labs, etc., students may not have seen any value in filling out the survey as compared to completing their other required educational endeavors. Furthermore, students may have chosen not to complete the survey as it would not directly benefit them. Although there have been many improvements addressing the issue of time constraints in medical education, additional reforms may still be needed to optimize time utilization for both medical students and their educators [25]. Associated with this limited number of surveys collected is self-selection: those students who did respond to the survey may not accurately reflect the population of the students in the class and the school. In fact, it has been shown that students who participate in education trials may be better students in several aspects [26]. Despite these limitations, all but one statistical parameter demonstrated a reasonable model fit: CFI fell just short of > 0.95 at 0.8623. On that basis, we believe that our conclusions are meaningful.

The decision to conduct the MSLQ survey immediately following the hyperammonemia session was based not so much on the subject matter but more so on the basis of its placement late in the series of active learning sessions involving the biochemistry faculty (Fig. 1, Table 1, and Appendix 1). Although we have added some hyperammonemia session-specific questions to the survey, the student characteristics identified should be applicable to other types of active learning sessions of Table 1 and Appendix 1. This notion is consistent with qualitative comments obtained from student evaluation of the various courses regarding active learning sessions. In this connection, it may be worthy to note that we have made attempts to determine whether the active learning session made a difference in student learning/retention of content by using “corresponding” questions (variations of the same question) in exams prior to (in the genitourinary course) and after (in the osteopathic patient care course) the hyperammonemia session. Preliminary analysis indicated no statistically significant difference in the % Correct in five such questions (ranging from 85 to 93 in % Correct). It should be noted, however, there is literature showing that passively obtained bits of information are easily lost over time [27, 28]. Moreover, our own studies on another active learning session (Appendix 1: V.-A. “Hey, Doc, can I safely eat this genetically modified salmon?”) suggest an attrition of knowledge on the general course concepts over a 1-month period [29]. The fact that we found knowledge on nitrogen metabolism and hyperammonemia to be retained (in that student performance was about the same within statistical norms from the first testing to the repeat testing 1 month later) suggests that the active learning exercise helped students in internalizing the concepts and transferring them from temporarily memorized ideas to longer-term application.

In conclusion, our study indicates students who perceive active learning sessions, such as the hyperammonemia session detailed in this report, to be an effective and desirable mode of learning possess certain characteristics (task value, intrinsic goal orientation, self-efficacy for learning and performance, control of learning beliefs, and effort regulation). These characteristics are likely a combination of the student’s innate attributes and the fostering of these attributes over the course of their educational development. Should we, as educators, encourage those traits that make active learning a “success,” or should we design modalities that better suit the students we have in medical schools? We believe that all students have growth potential; and thus, we believe that these characteristics can be acquired or enhanced such that all students gain the skills and knowledge provided to them through the active learning process. We also believe three levels of strategies are important in this endeavor: (a) communicating to incoming students, via institutional or curriculum leadership, how active learning skills can contribute to their becoming a successful physician; (b) deliberate introduction of active learning early in the curriculum and consistent use throughout the curriculum will help students’ confidence in their abilities to navigate the process; and (c) clear and advanced communication of learning objectives, session tasks, and knowledge gained for any particular active learning exercise.

## Supplementary Information

Below is the link to the electronic supplementary material.

Supplementary file1 (DOCX 47 kb)

Supplementary file2 (DOCX 30 kb)

Supplementary file3 (DOCX 52 kb)

Supplementary file4 (DOCX 32 kb)

Supplementary file5 (DOCX 40 kb)

## Data Availability

The datasets generated during and/or analyzed during the current study are available in the Harvard Dataverse repository, https://doi.org/10.7910/DVN/5AJPFE.
