# Supplementary material for: Understanding Student Characteristics in the Development of Active Learning Strategies
Source: Med Sci Educ. 2022 Apr 30;32(3):615–26. doi: 10.1007/s40670-022-01550-9 (PMC9270552; doi:10.1007/s40670-022-01550-9)
Supplement: Supplementary file 1 — Supplementary file1 (DOCX 47 kb) [file 40670_2022_1550_MOESM1_ESM.docx]

Seema Mehta^1^, Casey Schukow^1^, Amar Takrani^1^, Raquel Ritchie^2^, Carol Wilkins^3^, Martha Faner ^1^

^1^ Michigan State University, College of Osteopathic Medicine, Detroit Medical Center, Detroit, MI 48201

^2^ Michigan State University, College of Osteopathic Medicine, Macomb University Center, Clinton Twp, MI 48038

^3^ Michigan State University, College of Osteopathic Medicine, East Lansing, Michigan 48824

**Appendix 1. Information on Active Learning Activities Related to Biochemistry, Molecular Biology, and Genetics Content in the MSU-COM Pre-clinical Curriculum^a^**

^a^ The activities are listed, for the most part, in the order that students encounter them within the pre-clerkship curriculum. For each activity, the class is generally divided into six classrooms (~50 students/classroom with one faculty) and the students worked collaboratively in pods (~4 students/pod; ~12 pods/classroom).

**I. Metabolic Biochemistry**: 5 Activities

A. Electron Transport Chain

1. Preparatory work: Assigned reading on components, activities, and inhibitors of the Electron Transport Chain (ETC).

2. In-session Individual Readiness Quiz (IRQ): At the beginning of the session, students are assessed individually on 7 multiple-choice questions, using the i-Clicker® audience response system.

3. In-session Team-based Learning (TBL): Following the IRQ, students gather with their podmates and discuss the same 7 questions. Each pod then submits their collective response to each of the 7 questions using scratch cards that have a “star” marking the foil corresponding to the correct response. If the first scratch for a question fails to reveal a “star,” the pod can discuss the question again and attempt a second scratch, etc. until they find the “star.” After the session, the scratch card for each pod is graded on the basis of the number of attempts required to find the “star” for each question.

4. The number of points an individual student earns counting toward their Course grade represents the composite of the IRQ and TBL scores.

B. Unintended Consequences

1. Preparatory work: Students have completed the study of integration of carbohydrate and lipid metabolism. Assigned reading on water soluble vitamins and formative self-assessment on the assigned reading material.

2. At the beginning of the session, faculty presents the case: identification and chief complaint; history, physical exam, review of systems, and pertinent laboratory results including MRI imaging. [The case is a Wernicke’s encephalopathy due to gastric by-pass-induced vitamin B_1_ deficiency.]

3. Students then break-out into pods and analyze the case with instructions to answer a set of eight questions on the source, absorption, and function of vitamin B_1_, and the clinical manifestations of B_1_ deficiency particularly Wernicke’s encephalopathy. Three short review articles are provided to help answer the questions.

4. Students then reassemble as a class and share their answers to the eight questions.

5. Finally, students are assessed with multiple-choice questions (for points counting toward their Course grade) using the i-Clicker® audience response system.

C. Low Energy

1. Preparatory work: No specific assignment. Students have completed study of the integration of carbohydrate and lipid metabolism.

2. At the beginning of the session, faculty presents the patient case: identification and chief complaint; history of present illness; physical exam and review of systems; pertinent laboratory data. [The case is a medium-chain acyl CoA dehydrogenase (MCAD) manifested in an adult as rhabdomyolysis.]

3. Students then break-out into pods and discuss the case with instructions to address 11 specific items and ultimately to propose the likely cause of the patient’s symptoms in terms of: (a) the specific metabolic pathway affected; (b) the enzyme(s) that might be deficient; and (c) how the various observations of the patient and the lab results can be rationalized in the context of their hypothesis.

4. Students then reassemble as a classroom to answer multiple-choice questions (for points counting toward their Course grade) using the i-Clicker® audience response system.

5. The bulk of the in-session time is used for pods to share the answer(s) to the 11 specific items and discuss the most likely enzymatic defect/pathway.

D. What should be in the Differential?

1. Preparatory work: No specific assignment. Students have completed study of the integration of carbohydrate and lipid metabolism.

2. At the beginning of the session, faculty present patient case #1: identification and chief complaint; history of present illness; physical exam and review of systems; pertinent laboratory data. [Three cases of pediatric hypoglycemia (after fasting) with similar but distinct presentations due to deficiency in: (a) glucose 6-phosphatase (von Gierke disease); (b) glycogen phosphorylase (Hers disease); or (c) pyruvate carboxylase. Each session uses two of the three cases.]

3. Students then break-out into pods and discuss the case with instructions to list the top three possibilities in terms of an enzyme defect that would lead to the observed symptoms and laboratory results. Each pod submits their top three choices, and the submission is graded in terms of whether the correct enzyme defect is identified as the first choice, second choice, or third choice. If none of the choices are correct but one of the proposed possibilities is at least in the correct metabolic pathway, partial credit is given.

4. A second patient case is then presented, and the students again work in pods to propose their top three choices, using the same rationale. Their second submission is graded in the same way as the first. The number of points an individual student earns counting toward their Course grade represents the composite of the two scores.

5. Students then reassemble as a classroom and the faculty goes through the rationale at arriving at the top three enzymes that should be considered in the differential and how one particular enzyme defect best explains the observations made on the patients in the two cases.

E. Abnormality in Hormonal Regulation

1. Preparatory work: Assigned reading on diabetes mellitus and description of the patient case: identification and chief complaint; history of present illness, physical exam; pertinent laboratory data. [The case is on diabetic ketoacidosis.]

2. The session consists mainly of three rounds: (a) key aspects of the patient case; (b) review of the target tissues of insulin and glucagon and the key metabolic pathways and enzymes that are activated in each tissue when one hormone predominates; and (c) blood pH regulation in terms of ketoacidosis and respiratory compensation.

3. For each round, students first work in pods and then the class assembles as a whole to share thoughts and conclusions.

4. Finally, students are assessed with multiple-choice questions (for points counting toward their Course grade) using the i-Clicker® audience response system.

**II. Molecular Biology and Medical Genetics:** 2 Activities

A. Flipped classroom: cystic fibrosis (CF)

1. Preparatory work: Assigned reading on: (a) biochemistry of the Cystic Fibrosis Transmembrane Conductance Regulator (CFTR); (b) genetic aspects of CF; and (c) clinical manifestations as illustrated in a pediatric case. Students are assessed in terms of their preparatory work via an online Individual Readiness Quiz (IRQ).

2. This session takes place immediately prior to an exam in the Course and CF is used to review many aspects of the Course content up to this point (e.g. allelic heterogeneity; pleiotropy, variable expressivity, and population genetics of CF), using the i-Clicker® audience response system.

3. Participation in the Clicker response during the live session is required for eligibility to take a Post Exercise Quiz (PEQ). The number of points an individual student earns counting toward their Course grade represents the composite of the IRQ and PEQ scores.

4. This exercise on CF was developed to complement two other discussions on the condition in the curriculum: (a) a treatise on CF as a part of the coverage on epithelial membrane transport in pathophysiology; and (b) a direct interaction with a CF patient in terms of living with the condition as a part of osteopathic patient care.

B. Flipped classroom: approaches to correction of genetic disorders

1. This session follows a jigsaw model of teaching. Prior to the session, students/pods assigned to each classroom are divided into three groups; each group is responsible for learning about one approach to correction of a genetic disorder as illustrated with one specific example: (a) Group a --- correction at the protein level using enzyme replacement therapy (e.g. recombinant α-glucosidase infusion for Pompe disease); (b) Group b --- correction at the RNA level using antisense oligonucleotides to modulate splicing (e.g. antisense oligonucleotide treatment for Duchenne muscular dystrophy); (c) Group c --- correction at the DNA via delivery of the corrective gene (e.g. adeno-associated virus delivery of RPE65 for Leber congenital amaurosis).

2. Preparatory work: According to their Group assignment, each student will: (a) watch a mini-lecture (~15 min) that provides background to the genetic condition and to the corrective approach of the Group; (b) read journal article(s) corresponding to the Group; and (c) make a list of key points that the student will contribute to a 1-page primer that the student and their podmates would generate at the beginning of the live session; the primer represents an outline of key points that will be used to teach the classmates (assigned to other Groups) about the assigned topic. Each student will take an Individual Readiness Quiz (IRQ) corresponding to their Group assignment.

3. At the beginning of the live session, the members of a pod will compare their key point list and construct a 1-page primer to be used in teaching the Group’s condition and correction approach. Each pod will construct and submit one primer for grading.

4. Then, each pod in Group a will teach their content to neighboring pods in Group b and Group c. The class then assembles as a whole to answer multiple-choice questions using the i-Clicker® audience response system, each of which is followed by some discussion. The same process repeats itself: Group b pods teaching Group a and Group c, followed by Clicker questions and discussion; Group c pods teaching Group a and Group b, etc.

5. Participation in the Clicker response during the live session is required for eligibility to take a Post Exercise Quiz (PEQ). The number of points an individual student earns counting toward their Course grade represents the composite scores of the IRQ, PEQ, and 1-page primer submission.

**III. Genitourinary Systems Course:** 1 Activity

A. Newborn screening and phenylketonuria (PKU)

1. Preparatory work: No specific assignment. Session takes place toward the end of Semester 3 in the Year 1 curriculum; students have completed the basic sciences courses and the Neuromusculoskeletal system course and are in the midst of the Endocrine system and Genitourinary system courses.

2. The session consists of three parts: (a) Introductory scenario: a couple checks-in hospital for birth of a child and poses questions on the purpose of newborn screening. Working in pods, students use web resources to answer 9 questions on newborn screening (e.g. criteria for a condition to be included in the screening panel; how the panel varies state-by-state, etc.). Students reassemble as a whole classroom and share their findings by responding to the questions as if they were the physician addressing the patient; this is followed by some multiple-choice questions using the i-Clicker® audience response system to confirm understanding. (b) Faculty provides information on basic steps of the newborn screening procedure (e.g. video on blood sample collection; principal methods on screening assays, including mass spectrometry). (c) Scenario on results of the newborn screening yielding a positive finding for PKU. Working in pods, students use web resources to answer 5 questions on how to inform the parents (e.g. what screening results mean and how to obtain diagnostic tests). Students then reassemble as a whole classroom and share their findings. This is followed by Clicker questions and a discussion of PKU, including the importance of follow-up and the consequences of maternal PKU syndrome.

3. Participation in the Clicker response during the live session is required for eligibility to take a Post Exercise Quiz (PEQ).

**IV. Ethics, Professionalism and Law:** 1 Activity

A. Trey fell off the couch

1. Preparatory work: No specific assignment. Students are essentially at the end of their pre-clerkship curriculum.

2. This session represents the lone exception to the general procedure of breaking the class into six separate classrooms. The class remains as a whole with the three campuses connected via Polycom video conferencing.

3. At the beginning of the session, faculty presents the patient case: identification and chief complaint; history of present illness; physical exam and review of systems; pertinent laboratory data. [The case is a Glutaric Aciduria type 1, initially misdiagnosed, at 15-month of age, as non-accidental trauma because the patient presented with retinal hemorrhage and subdural hematoma. The case description, laboratory data and the associated video recording were developed by Dr. James D. Shoemaker, Department of Biochemistry, St. Louis University School of Medicine, who kindly shared this teaching material with us.]

4. Students are given some time to discuss with their immediate neighbors, to search online resources and to come up with a list of their top three possible diagnoses.

5. This is followed by a video of an interview with the parents, revealing new information when their son Trey, now 15-years-old, underwent a sports physical. The astute physician ordered a series of tests to further investigate what might have happened when Trey was a toddler.

6. Students are then given time to discuss the case with the new information (mass spectrometry data) and the question: How does this change your list of top three diagnoses?

6. Students reassemble to share their thoughts, with discussion on the nuances of the diagnostic process and how misdiagnosis may affect patients and their families.

**V. Osteopathic Patient Care:** 2 Activities

A. “Hey, Doc, can I safely eat this genetically modified salmon?”

1. Preparatory work: Assigned reading on the background of the AquAdvantage salmon, with instructions to answer 11 questions on the recombinant DNA technology involved in generating the genetically modified salmon and the biochemistry relevant in comparing the compositions of amino acid, carbohydrate, lipid, and vitamins, after digestion and absorption of an identically sized filet of wild-type and genetically modified fish. The session takes place in a longitudinal patient care course in the context of answering a question from the patient; students have just completed the coverage of molecular biology in a parallel basic science course.

2. The session begins with Individual Readiness Quiz (IRQ) using the i-Clicker® audience response system. Each question is discussed in detail, with active student input, so that key elements of the biochemistry and molecular biology are covered.

3. “Hey, Doc, can I safely eat this genetically modified salmon?” is then posed in the context of a patient’s question after a routine office visit. Students then break-out into pods and discuss how to articulate a response to the patient. Each pod submits a written response that is graded following a rubric. The number of points an individual student earns counting toward their Course grade represents the composite scores of the IRQ and their pod submission.

4. Students then reassemble as a class and share their submissions.

5. For more information, please see J.M. Simmons and R.P. Ritchie (2017) “Training Students to Answer Layman’s Questions Also Helps in Retention of Scientific Content,” Med. Sci. Educ. **27**: 33-39.

B. Hyperammonemia

1. This activity is described in the body of the present manuscript.
