# Supplementary material for: Understanding Student Characteristics in the Development of Active Learning Strategies
Source: Med Sci Educ. 2022 Apr 30;32(3):615–26. doi: 10.1007/s40670-022-01550-9 (PMC9270552; doi:10.1007/s40670-022-01550-9)
Supplement: Supplementary file 2 — Supplementary file2 (DOCX 30 kb) [file 40670_2022_1550_MOESM2_ESM.docx]

Seema Mehta^1^**^†^**, Casey Schukow^1^**^†^**, Amar Takrani^1^**^†^**, Raquel Ritchie^2^, Carol Wilkins^3^, Martha Faner ^1*^

^1^ Michigan State University, College of Osteopathic Medicine, Detroit Medical Center, Detroit, MI 48201

^2^ Michigan State University, College of Osteopathic Medicine, Macomb University Center, Clinton Twp, MI 48038

^3^ Michigan State University, College of Osteopathic Medicine, East Lansing, Michigan 48824

**Appendix 2. Modified MSLQ Questionnaire**

The following questions ask about your attitudes towards the evidence based medicine on hyperammonemia session that you just took part in. **Remember there are no right or wrong answers, just answer as accurately as possible**. Use the scale below to answer the questions. If you think the statement is very true of you, circle 7; if a statement is not at all true of you, circle 1. If the statement is more or less true of you, find the number between 1 and 7 that best describes you.

1 2 3 4 5 6 7

not at all true of me very true of me

1. This session has improved my ability to search for relevant journal articles.
2. This session reinforced important concepts on the topic of nitrogen metabolism (ammonia and urea) previously introduced in OST 572, Genitourinary System.
3. This session improved my understanding of how basic science content (urea cycle) forms the basis for clinical application (hyperammonemia).
4. Reading the gold standard article as a team was an efficient way to learn the necessary information.
5. Writing a summary as a team helped me in understanding content as well as in developing clarity of thought.
6. I felt a greater sense of social support from my classmates during this session than in a traditional lecture.
7. In general, I feel an active learning session (like the one I experienced today) is a more effective way than lectures to learn the required material.
8. In general, I would be more inclined to attend class in person if the time was used for group activities or problem solving rather than a lecture presentation.

**THE MOTIVATED STRATEGIES FOR LEARNING QUESTIONNAIRE**

**Part A. Motivation**

The following questions ask about your motivation for and attitudes about the Michigan State University College of Osteopathic Medicine (MSUCOM) curriculum in general. **Remember there are no right or wrong answers, just answer as accurately as possible**. Use the scale below to answer the questions. If you think the statement is very true of you, circle 7; if a statement is not at all true of you, circle 1. If the statement is more or less true of you, find the number between 1 and 7 that best describes you.

1 2 3 4 5 6 7

not at all true of me very true of me

1. In this curriculum, I prefer course materials that really challenge me so I can learn new things.
2. If I study in appropriate ways, then I will be able to learn the material in this curriculum.
3. When I take a test I think about how poorly I am doing compared with other students.
4. In this curriculum, I think I will be able to use what I learn in one course in other courses.
5. I believe I will receive excellent grades in this curriculum.
6. I'm certain I can understand the most difficult material in this curriculum.
7. Getting a good grades in this curriculum is the most satisfying thing for me right now.
8. When I take a test I think about items on other parts of the test I can't answer.
9. It is my own fault if I don’t learn the materials in this curriculum.
10. It is important for me to learn the course materials in this curriculum.
11. The most important thing for me right now is improving my overall chances of a desirable residency match, so my main concern in this curriculum is getting good grades.
12. I'm confident I can learn the basic concepts taught in this curriculum.
13. If I can, I want to get better grades in this curriculum than most of the other students.
14. When I take tests I think of the consequences of failing.
15. I'm confident I can understand the most complex material presented by the instructors in this curriculum.
16. In a curriculum like this, I prefer course materials that arouse my curiosity, even if they are difficult to learn.
17. I am very interested in ALL the content area of this curriculum.
18. If I try hard enough, then I will understand the course materials.
19. I have an uneasy, upset feeling when I take an exam.
20. I'm confident I can do an excellent job on the assignments and tests in this curriculum.
21. I expect to do well in this curriculum.
22. The most satisfying thing for me in this curriculum is trying to understand the content as thoroughly as possible.
23. I think the course materials in this curriculum will be useful for me to learn.
24. If given the opportunity in this curriculum, I would choose course assignments that I can learn from even if they don't guarantee a good grade.
25. If I don't understand the material in the curriculum, it is because I didn't try hard enough.
26. I like the subject matter of this curriculum.
27. Understanding the subject matter of this curriculum is very important to me.
28. I feel my heart beating fast when I take an exam.
29. I'm certain I can master the skills being taught in this curriculum.
30. I want to do well in this curriculum because it is important to show my ability to prospective residency directors.
31. Considering the difficulty of this curriculum, the teachers, and my skills, I think I will do well in this curriculum.

**Part B. Learning Strategies**

The following questions ask about your learning strategies and study skills for the Michigan State University College of Osteopathic Medicine (MSUCOM) curriculum in general. **Again, there are no right or wrong answers. Answer the questions about how you study in this class as accurately as possible**. Use the same scale to answer the remaining questions. If you think the statement is very true of you, circle 7; if a statement is not at all true of you, circle 1. If the statement is more or less true of you, find the number between 1 and 7 that best describes you.

1 2 3 4 5 6 7

not at all true of me very true of me

1. When I study the readings for this curriculum, I outline the material to help me organize my thoughts.
2. During class time I often miss important points because I'm thinking of other things.
3. When studying for this curriculum, I often try to explain the material to a classmate or friend.
4. I usually study in a place where I can concentrate on my course work.
5. When reading for the courses in this curriculum, I make up questions to help focus my reading.
6. I often feel so lazy or bored when I study for my courses that I quit before I finish what I planned to do.
7. I often find myself questioning things I hear or read in this curriculum to decide if I find them convincing.
8. When I study for this curriculum, I practice saying the material to myself over and over.
9. Even if I have trouble learning the material in this curriculum, I try to do the work on my own, without help from anyone.
10. When I become confused about something I'm reading for this curriculum, I go back and try to figure it out.
11. When I study for my courses, I go through the readings and my class notes and try to find the most important ideas.
12. I make good use of my study time for my courses.
13. If course readings are difficult to understand, I change the way I read the material.
14. I try to work with my classmates to complete the course assignments.
15. When studying for my courses, I read my class notes and the course readings over and over again.
16. When a theory, interpretation, or conclusion is presented in class or in the readings, I try to decide if there is good supporting evidence.
17. I work hard to do well in my courses even if I don't like what we are doing.
18. I make simple charts, diagrams, or tables to help me organize course material.
19. When studying for my courses, I often set aside time to discuss course material with a group of students from the class.
20. I treat the course material as a starting point and try to develop my own ideas about it.
21. I find it hard to stick to a study schedule.
22. When I study for my courses, I pull together information from different sources, such as lectures, readings, and discussions.
23. Before I study new course material thoroughly, I often skim it to see how it is organized.
24. I ask myself questions to make sure I understand the material I have been studying in my courses.
25. I try to change the way I study in order to fit the course requirements and the instructor's teaching style.
26. I often find that I have been reading for my courses but don't know what it was all about.
27. I ask the instructor to clarify concepts I don't understand well.
28. I memorize key words to remind me of important concepts in my courses.
29. When course work is difficult, I either give up or only study the easy parts.
30. I try to think through a topic and decide what I am supposed to learn from it rather than just reading it over when studying for my courses.
31. I try to relate ideas in the subject of one course to those in other courses whenever possible.
32. When I study for my courses, I go over my class notes and make an outline of important concepts.
33. When reading for my courses, I try to relate the material to what I already know.
34. I have a regular place set aside for studying.
35. I try to play around with ideas of my own related to what I am learning in my courses.
36. When I study for my courses, I write brief summaries of the main ideas from the readings and my class notes.
37. When I can't understand the material in my courses, I ask another student in my class for help.
38. I try to understand the material in my courses by making connections between the readings and the concepts from the lectures.
39. I make sure that I keep up with the weekly readings and assignments for my courses.
40. Whenever I read or hear an assertion or conclusion in my courses, I think about possible alternatives.
41. I make lists of important items for my courses and memorize the lists.
42. I attend class in person regularly.
43. Even when course materials are dull and uninteresting, I manage to keep working until I finish.
44. I try to identify students in my courses whom I can ask for help if necessary.
45. When studying for my courses I try to determine which concepts I don't understand well.
46. I often find that I don't spend very much time on my course work because of other activities.
47. When I study for my courses, I set goals for myself in order to direct my activities in each study period.
48. If I get confused taking notes in class, I make sure I sort it out afterwards.
49. I rarely find time to review my notes or readings before an exam.
50. I try to apply ideas from course readings in other class activities such as lecture and discussion.
