# Supplementary material for: Understanding Student Characteristics in the Development of Active Learning Strategies
Source: Med Sci Educ. 2022 Apr 30;32(3):615–26. doi: 10.1007/s40670-022-01550-9 (PMC9270552; doi:10.1007/s40670-022-01550-9)
Supplement: Supplementary file 3 — Supplementary file3 (DOCX 52 kb) [file 40670_2022_1550_MOESM3_ESM.docx]

Seema Mehta^1^, Casey Schukow^1^, Amar Takrani^1^, Raquel Ritchie^2^, Carol Wilkins^3^, Martha Faner ^1^

^1^ Michigan State University, College of Osteopathic Medicine, Detroit Medical Center, Detroit, MI 48201

^2^ Michigan State University, College of Osteopathic Medicine, Macomb University Center, Clinton Twp, MI 48038

^3^ Michigan State University, College of Osteopathic Medicine, East Lansing, Michigan 48824

**Appendix 3:** Nine factors predicted by the EFA and associated pattern coefficients. Item number as well as the subscale that the item was designed to test is noted. Only items with a pattern coefficient of >0.4 are included and pattern coefficients of <0.4 are not shown.

| Items | **Factors** | | | | | | | | |
| --- | --- | --- | --- | --- | --- | --- | --- | --- | --- |
|  | **I** | **II** | **III** | **IV** | **V** | **VI** | **VII** | **VIII** | **IX** |
| 10 (control of learning beliefs) |  | 0.479 |  |  |  |  |  |  |  |
| 11 (test anxiety) |  |  |  |  |  | 0.554 |  |  |  |
| 13 (self-efficacy for learning and performance) |  | 0.473 |  |  | 0.563 |  |  |  |  |
| 14 (self-efficacy for learning and performance) |  | 0.511 |  |  | 0.508 |  |  |  |  |
| 15 (extrinsic goal orientation) |  |  |  |  |  | 0.571 |  |  |  |
| 16 (test anxiety) |  |  |  |  |  | 0.521 |  |  |  |
| 17 (control of learning beliefs) |  | 0.480 |  |  |  |  |  |  |  |
| 18 (task value) |  | 0.449 |  |  |  |  |  |  |  |
| 19 (extrinsic goal orientation) |  |  |  |  |  | 0.560 |  |  |  |
| 20 (self-efficacy for learning and performance) |  | 0.488 |  |  | 0.479 |  |  |  |  |
| 22 (test anxiety) |  |  |  |  |  | 0.607 |  |  |  |
| 23 (self-efficacy for learning and performance) |  | 0.616 |  |  | 0.462 |  |  |  |  |
| 24 (intrinsic goal orientation) |  | 0.523 |  |  |  |  |  |  |  |
| 26 (control of learning beliefs) |  | 0.525 |  |  | 0.481 |  |  |  |  |
| 27 (test anxiety) |  |  |  |  |  | 0.667 |  |  |  |
| 28 (self-efficacy for learning and performance) |  | 0.507 |  |  | 0.642 |  |  |  |  |
| 29 (self-efficacy for learning and performance) |  | 0.507 |  |  | 0.701 |  |  |  |  |
| 31 (task value) |  | 0.528 |  |  |  |  |  |  |  |
| 32 (intrinsic goal orientation) |  | 0.406 |  |  |  |  |  |  |  |
| 33 (control of learning beliefs) |  | 0.663 | 0.579 |  |  |  |  |  |  |
| 34 (task value) |  | 0.762 |  |  |  |  |  |  |  |
| 35 (task value) |  | 0.529 |  |  |  |  |  |  |  |
| 37 (self-efficacy for learning and performance) |  | 0.665 |  |  | 0.417 |  |  |  |  |
| 38 (extrinsic goal orientation) |  |  |  |  |  | 0.404 |  |  |  |
| 39 (self-efficacy for learning and performance) |  | 0.610 |  |  | 0.480 |  |  |  |  |
| 40 (organization) | 0.495 |  | 0.450 |  |  |  |  |  |  |
| 43 (time and study environment) |  | 0.402 |  |  |  |  |  |  |  |
| 46 (critical thinking) |  |  |  |  |  |  | 0.411 |  |  |
| 49 (meta-cognitive self-regulation) |  | 0.433 |  |  |  |  |  |  |  |
| 51 (time and study environment) |  |  |  |  | 0.412 |  |  |  |  |
| 53 (peer learning) | 0.775 |  |  |  |  |  |  |  |  |
| 54 (rehearsal) |  |  |  |  |  |  |  | 0.448 |  |
| 55 (critical thinking) |  |  |  |  |  |  | 0.477 |  |  |
| 56 (effort regulation) |  |  |  |  | 0.445 |  |  |  |  |
| 58 (peer learning) | 0.523 |  |  |  |  |  |  |  |  |
| 59 (critical thinking) | 0.476 |  |  |  |  |  |  |  |  |
| 60 (time and study) |  |  |  |  |  |  |  |  |  |
| 63 (meta-cognitive self-regulation) | 0.473 |  |  |  |  |  |  |  |  |
| 64 (meta-cognitive self regulation) |  |  |  |  |  |  | 0.481 |  |  |
| 68 (effort regulation) |  |  |  |  |  |  |  | 0.499 |  |
| 71 (organization) | 0.686 |  | 0.587 | 0.406 |  |  |  |  |  |
| 72 (elaboration) |  |  |  |  |  |  | 0.409 |  |  |
| 73 (time and study environment) |  |  |  |  | 0.527 |  |  |  |  |
| 74 (critical thinking) |  |  |  |  |  |  | 0.411 |  |  |
| 75 (elaboration) |  |  | 0.448 |  |  |  |  |  |  |
| 76 (help seeking) | 0.406 |  |  |  |  |  |  | 0.435 |  |
| 77 (elaboration) | 0.465 |  |  |  |  |  |  |  |  |
| 79 (critical thinking) | 0.432 |  |  |  |  |  | 0.471 |  |  |
| 82 (effort regulation) |  |  |  |  |  |  |  | 0.404 |  |
| 84 (meta-cognitive self-regulation) |  |  |  |  |  |  |  |  | 0.664 |
| 86 (meta-cognitive self-regulation) |  |  |  |  |  |  |  |  | 0.567 |
| 87 (meta-cognitive self-regulation) |  |  |  |  |  |  |  |  | 0.437 |
| 88 (time and study) |  |  |  |  |  |  |  | 0.473 |  |
| 89 (elaboration) |  |  |  |  |  |  |  |  | 0.428 |
