# Supplementary material for: Understanding Student Characteristics in the Development of Active Learning Strategies
Source: Med Sci Educ. 2022 Apr 30;32(3):615–26. doi: 10.1007/s40670-022-01550-9 (PMC9270552; doi:10.1007/s40670-022-01550-9)
Supplement: Supplementary file 4 — Supplementary file4 (DOCX 32 kb) [file 40670_2022_1550_MOESM4_ESM.docx]

Seema Mehta^1^, Casey Schukow^1^, Amar Takrani^1^, Raquel Ritchie^2^, Carol Wilkins^3^, Martha Faner ^1^

^1^ Michigan State University, College of Osteopathic Medicine, Detroit Medical Center, Detroit, MI 48201

^2^ Michigan State University, College of Osteopathic Medicine, Macomb University Center, Clinton Twp, MI 48038

^3^ Michigan State University, College of Osteopathic Medicine, East Lansing, Michigan 48824

**Appendix 4.** Confirmatory Factor Analysis indices and standard regression weights for each of the seven factors delineated by Exploratory Factor Analysis**^a^**

|  | **TV** | **SELP** | **CLB** | **TA** | **EGO** | **CT** | **MSR** |
| --- | --- | --- | --- | --- | --- | --- | --- |
| *Cronbach’s Alpha***^b^** | .832 | .936 | .731 | .827 | .756 | .750 | .713 |
| **Measure of Covariance^c^** | | | | | | | |
| *TV* |  |  |  |  |  |  | .406 |
| *SELP* | .680 |  |  |  |  |  | .334 |
| *CLB* | .717 | .833 |  |  |  |  | .394 |
| *TA* | .024 | *-.248*  *(p = 0.02)* | *-.175*  *(p = 0.049)* |  |  |  | .049 |
| *EGO* | .602 | .432 | .422 | .301 |  |  | .330 |
| *CT* |  | .255 | .165 | .089 | .585 |  | .160 |

**^a^**The seven factors: TV= task value; SELP= self-efficacy for learning and performance; CLB= control of learning beliefs; TA= test anxiety; EGO= extrinsic goal orientation; CT= critical thinking; MSR= meta-cognitive self-regulation.

**^b^**Coefficient (Cronbach’s) Alpha measures internal consistency.

**^c^**Covariance/Correlations of the seven factors with items of the dataset. P values given for statistically significant co-variances.
