# Supplementary material for: Understanding Student Characteristics in the Development of Active Learning Strategies
Source: Med Sci Educ. 2022 Apr 30;32(3):615–26. doi: 10.1007/s40670-022-01550-9 (PMC9270552; doi:10.1007/s40670-022-01550-9)
Supplement: Supplementary file 5 — Supplementary file5 (DOCX 40 kb) [file 40670_2022_1550_MOESM5_ESM.docx]

Seema Mehta^1^, Casey Schukow^1^, Amar Takrani^1^, Raquel Ritchie^2^, Carol Wilkins^3^, Martha Faner ^1^

^1^ Michigan State University, College of Osteopathic Medicine, Detroit Medical Center, Detroit, MI 48201

^2^ Michigan State University, College of Osteopathic Medicine, Macomb University Center, Clinton Twp, MI 48038

^3^ Michigan State University, College of Osteopathic Medicine, East Lansing, Michigan 48824

**Appendix 5.** Pearson coefficients and strengths of association for the seven-factor model and associated 8-item questionnaire variables.

| **Factors** | **Q1** | **Q2** | **Q3** | **Q4** | **Q5** | **Q6** | **Q7** | **Q8** |  |
| --- | --- | --- | --- | --- | --- | --- | --- | --- | --- |
| **1 (TV)** | 0.35 | 0.30 | 0.37 | 0.25 | 0.32 | 0.22 | 0.34 | 0.19 |  |
| **2 (SELP)** | 0.39 | 0.30 | 0.32 | 0.19 | 0.23 | 0.22 | 0.13 | 0.06 |  |
| **3 (CLB)** | 0.37 | 0.25 | 0.32 | 0.25 | 0.20 | 0.16 | 0.08 | 0.02 |  |
| **4 (TA)** | -0.01 | -0.06 | -0.06 | 0.08 | 0.06 | 0.00 | 0.08 | 0.14 |  |
| **5 (EGO)** | 0.28 | 0.11 | 0.14 | 0.13 | 0.13 | 0.14 | 0.08 | 0.16 |  |
| **6 (CT)** | 0.14 | 0.16 | 0.17 | 0.01 | 0.14 | 0.12 | 0.16 | 0.18 |  |
| **7 (MSR)** | 0.15 | 0.09 | 0.15 | 0.02 | 0.00 | 0.02 | -0.04 | -0.03 |  |
| **Q9 (IGO)** | 0.37 | 0.37 | 0.43 | 0.32 | 0.36 | 0.34 | 0.42 | 0.36 |  |
| **Q12 (TV)** | 0.37 | 0.24 | 0.33 | 0.29 | 0.26 | 0.25 | 0.29 | 0.19 |  |
| **Q30 (IGO)** | 0.37 | 0.22 | 0.33 | 0.23 | 0.31 | 0.19 | 0.29 | 0.18 |  |
| **Q32 (IGO)** | 0.32 | 0.09 | 0.16 | 0.25 | 0.28 | 0.20 | 0.25 | 0.17 |  |
| **Q56 (ER)** | 0.21 | 0.26 | 0.31 | 0.11 | 0.14 | 0.09 | 0.20 | 0.18 |  |
| **Legend:** Shaded cells have Pearson coefficients between 0.3-0.5. All shaded cells have an associated p-value of <0.05. TV = Task value; SELP = Self-efficacy for learning & performance; CLB = Control of learning beliefs; TA = Test anxiety; EGO = Extrinsic goal orientation; CT = Critical thinking; MSR = Meta-cognitive self-regulation; IGO = Intrinsic Goal Orientation; ER = Effort Regulation | | | | | | | | | |
